# Medical Costs and Caregiver Burden of Delivering Disease-Modifying Alzheimer’s Treatments with Different Duration and Route of Administration

**DOI:** 10.14283/jpad.2024.81

**Published:** 2024-05-07

**Authors:** T. Ozawa, G. Franguridi, Soeren Mattke

**Affiliations:** 1 The USC Brain Health Observatory, University of Southern California, Los Angeles, CA USA; 2 The USC Brain Health Observatory, USC Dornsife, 635 Downey Way, #505N, Los Angeles, CA 90089 USA

**Keywords:** Alzheimer’s disease, disease modifying treatment, dementia, economic evaluation

## Abstract

**Background:**

Multiple disease modifying treatment for Alzheimer’s disease are currently in clinical development or have been recently approved for use. They have vastly different treatment properties but so far, little work has been done to quantify the impact of treatment properties on the treatment’s value in terms of medical and social care costs and caregiver burden.

**Objectives:**

This study aims to analyze how the mode of treatment administration, treatment frequency and duration, and monitoring requirements affect the value of disease modifying treatments. In order to isolate these effects, we compare five hypothetical disease modifying treatments with equal efficacy and safety: (1) chronic bi-weekly intravenous infusion, (2) chronic four-weekly intravenous infusion, (3) 52 weeks fixed duration four-weekly intravenous infusion, (4) chronic subcutaneous injections, and (5) chronic oral prescription on their direct medical costs, caregiver burden, and preservation of treatment value.

**Design:**

Survey of Alzheimer’s disease treatment clinics and retrospective data analysis.

**Setting:**

United States.

**Measurements:**

Direct medical cost and caregiver burden of treatment administration and monitoring compared to gross treatment benefit.

**Results:**

Chronic bi-weekly infusion treatment had the highest direct medical cost ($45,208) and caregiver burden ($6,095), reducing the treatment value by 44%, while oral treatment with the lowest direct medical cost ($1,983) and caregiver burden ($457) reduced the treatment value by only 2%. Substantial caregiver burden was reported from the survey, with a reported average of 2.3 hours for an office visit and infusion, 44 minutes of round-trip travel time, and 78% of patients being accompanied by a caregiver for treatment.

**Conclusion:**

Burden of chronic intravenous treatments exceed the gross medical and social care cost savings and value of caregiver benefit. The results suggest the need for less complex treatments that require fewer clinic visits to preserve the economic value of disease modifying treatments.

## Introduction

**M**ultiple disease modifying treatments (DMTs) for Alzheimer’s disease (AD) are in late-stage clinical development or have been approved for use (1). These DMTs differ greatly in their mechanism of action, mode of administration, treatment frequency and duration, and monitoring requirements.

The two drugs currently approved by the U.S. Food and Drug Administration (FDA) for the treatment of early AD, aducanumab and lecanemab, are monoclonal antibodies that remove amyloid deposits from the brain. They require in-clinic intravenous administration four weekly and biweekly, respectively, for an unspecified duration (2, 3) and a subcutaneous formulation of lecanemab is currently being tested. By contrast, donanemab is a fixed-duration amyloid targeting antibody that is delivered intravenously every four weeks for up to 72 weeks. The treatment can be ended earlier at 24 weeks or 52 weeks if amyloid clearance is achieved and follows a similar monitoring protocol as aducanumab and lecanemab (4). All amyloid-targeting treatments require at least four MRIs in the first year of treatment to monitor for Amyloid-Related Imaging Abnormalities (ARIA), which refers to the emergence of cerebral edema or microhemorrhages. ARIA typically causes no or limited symptoms, such as headache and nausea, which resolve with temporary treatment interruption. However, severe cases with subsequent mortality have been reported (5). In addition, the oral drugs valiltramiprosate and semaglutide, which have different modes of action and no known ARIA risk, are being studied in phase 3 trials. Valiltramiprosate inhibits the aggregation of soluble in the brain and thereby plaque formation (6). Semaglutide is a Glucagon-like peptide-1 receptor agonists that is indicated for type 2 diabetes and obesity and is believed to have positive metabolic and anti-inflammatory effects in the brain. (7)

The varying treatment properties lead to substantially different treatment burdens in terms of direct medical cost and caregiver burden. One way to lower the treatment burden is by limiting the duration of treatment. A recent study by Boustani et al. quantified the cost-effectiveness of time-limited treatments, comparing the cost-effectiveness of hypothetical DMTs with continuous (treatment until progression to severe AD), fixed duration (18 months), and test-and-discontinue (40% ending treatment at 6 months) treatment strategies (8). Assuming an annual drug price of $56,000, they found that relative to continuous treatment, the incremental cost-effectiveness ratio (ICER) decreased significantly by 74% for the fixed duration and 79% for the test-and-discontinue strategies.

In addition to limiting treatment duration, another way to lower the treatment burden is by reducing the complexity of treatment administration and monitoring. Subcutaneous and oral drugs like semaglutide and valiltramiprosate can be administered at the patient’s home, resulting in less costly and burdensome treatment administration, and the two oral compounds also have less complex monitoring protocols than the amyloid-targeting antibodies (9, 10). Reducing treatment complexity could substantially lower the burden on family caregivers, many of whom arrange and provide transportation to medical appointments, even for patients in the early stages of symptomatic AD (11–13).

So far, little work has been done to quantify the impact of DMT properties besides treatment duration on the treatment burden and the extent to which they preserve the value of treatments. Therefore, this study aims to analyze how the mode of treatment administration, treatment frequency and duration, and monitoring requirements affect the value of hypothetical DMTs in terms of medical costs and caregiver burden.

## Methods

### Treatment properties and protocol for administration and monitoring

We estimated the impact of treatment burden on the treatment value of five hypothetical DMTs (See Table 1). The five hypothetical treatments are (1) chronic bi-weekly intravenous infusion, (2) chronic four-weekly intravenous infusion, (3) 52 weeks fixed duration four-weekly intravenous infusion, (4) chronic subcutaneous injections, and (5) chronic oral prescription.

**Table 1:** Description of the five treatment scenarios

**Treatment Scenario**	**Treatment Mode**	**Treatment Duration**	**Treatment Weeks**	**Treatment Frequency**	**Location**
1	Intravenous infusion	Chronic	376	Bi-weekly	Clinic
2	Intravenous infusion	Chronic	376	Four-weekly	Clinic
3	Intravenous infusion	Fixed	52	Four-weekly	Clinic
4	Subcutaneous injection	Chronic	376	Four-weekly	Patient home
5	Oral prescription	Chronic	376	Daily	Patient home

To be able to isolate the impact of differential drug properties on the treatment burden, we held the treatment efficacy and safety properties constant. We assumed that treatment begins at mild cognitive impairment (MCI) and reduces disease progression by 30%, with a 25% ARIA risk in the first year for injectable agents. For chronic treatments, we assumed that treatment continues until patients progress to moderate dementia, which takes 7.24 years on average (14). For the fixed-duration treatment, patients are retreated once in year 5 for amyloid reaccumulation.

We based monitoring on appropriate use recommendations for lecanemab (3), assuming four MRIs in the first year for all injectable treatments and then once a year until the end of treatment duration. As the oral drug was assumed to have no ARIA risk, it did not require MRI monitoring. In addition, we assumed that 25% of patients, who experience ARIA, require three additional MRIs. Quarterly monitoring office visits were added for the fixed-duration treatments once infusion delivery was completed and for the oral drug for the entire treatment duration. The fixed-duration treatment requires monitoring with two PET scans to ascertain amyloid clearance, and subsequently annual blood tests for the recurrence of amyloid pathology.

### Gross treatment benefit

Gross treatment benefit was obtained from a prior publication by Prados et al. (15), who calculated the societal value of a hypothetical AD treatment, starting at the MCI stage, and delaying disease progression by 30%. The societal value includes the patient’s avoided direct medical cost, community social care and nursing home costs, and gains in QALY compared to natural disease progression, as well as the caregiver’s direct medical cost, lost productive and leisure hours, and QALY gains. QALY gains were monetized at $150,000 per QALY, and caregiver time was monetized using the April 2023 US average hourly wage for lost productive hours, and 35% of hourly wage for leisure hours (16). All other values from the publication were also inflated to 2023 USD, resulting in an estimated direct cost reduction of $23,933, patient QALY gains of $87,186, and reduced caregiver burden of $4,256.

### Data sources

#### Direct medical cost

Direct medical costs were based on the 2023 Medicare payment rates and inflated by 3% annually for future years using the following CPT and HCPCS codes: brain MRI without contrast (70551/$226.20), office visit (99212/$62.20), intravenous infusion delivery (96413/$144.39), PET scan of the brain (78814/$1,570), amyloid tracer (A9586/$3,355). The cost of an AD blood test was obtained from a published study ($540) (17).

#### Caregiver burden

Caregiver burden was operationalized as their time spent accompanying patients to clinic visits for treatment delivery and monitoring, including driving time. Valuation of that time use followed the approach taken by Gustavsson et al. (18), who reported that the ratio of lost productive to lost leisure time for caregivers of AD patients was approximately 1:52 and valued lost leisure at 35% of lost productive time. We used the 2023 US average gross wage of $33.36/hour, inflated by 3% in each future year, as an estimate for the value of a productive hour.

Caregiver time use was generated from a survey of a convenience sample of 42 AD treatment sites with a 67% (n=28) response rate. We asked sites to estimate the average duration of treatment and monitoring visits, including time spent for registration and waiting, round trip travel time to the clinic, and the average proportion of patients accompanied by a caregiver. Following lecanemab’s monitoring protocol, we added observation times of three hours following the first infusion and two hours for the second and third infusions to the time use estimates (3).

#### Net treatment value calculation

As outlined above, the gross value of the five treatments was assumed to be identical, and the contribution of patient QALY gains to that value was assumed to be unaffected by the different treatment properties. The remaining net value specific to each hypothetical treatment was calculated as the difference of gross value and direct medical cost and caregiver burden of administration and monitoring.

#### Sensitivity analysis

We conducted a probabilistic sensitivity analysis to illustrate how the uncertainty in our input parameters for benefit and cost of each of the five treatment modalities influences the estimate of their net value. Uncertainty in gross benefit was derived from the aforementioned study by Prados et al., who had included estimates for treatment effect sizes of 22% and 40% in addition to the base case estimate of 30%. We assumed that medical cost for administration and monitoring varied +/− 10% and used the variance in the time use estimates for uncertainty in caregiver burden estimates. We repeated the analysis 1,000 times, drawing each time parameters from their underlying distribution at random. Treatment effect sizes and medical cost were assumed to be distributed uniformly and caregiver time use normally.

## Results

### Direct medical cost

The direct medical cost for administration and monitoring of the five treatments are summarized in Table 2. The treatment with chronic bi-weekly infusion would have the highest direct medical cost of $45,208 due to the need for 188 clinic visits, resulting in cost of $29,792 (66%), $12,834 (28%) $2,581 (6%) for infusion delivery, evaluations, and MRI monitoring, respectively. With 93 clinic visits, a treatment requiring infusions every four weeks would reduce the direct medical cost by about half (52%).

**Table 2:** Direct medical cost and caregiver burden of treatments

	**Chronic Biweekly**	**Chronic 4-weekly**	**Fixed 52 weeks**	**Subcutaneous**	**Oral**
Direct medical cost	$45,208	$23,660	$17,319	$4,502	$1,983
Infusion	66%	62%	9%	0%	0%
Examination	28%	27%	14%	43%	100%
Monitoring	6%	11%	77%	57%	0%
Caregiver burden	$6,095	$3,143	$914	$613	$457

While a treatment administered every four weeks for 52 weeks would have 88% fewer infusions and 62% fewer office visits than a chronic treatment with same administration frequency, total direct medical cost would only be 27% lower because of the added cost of monitoring for initial and sustained amyloid clearance. The need for additional PET scans and blood tests would account for $12,178 in additional medical cost.

Treatments for subcutaneous injection and oral application would have 10% and 4%, respectively, of the direct medical cost of a treatment for chronic use and bi-weekly application and 26% and 11% of that for the fixed duration treatment. The difference between the subcutaneous and the oral treatment is due to the higher monitoring burden with 11 MRIs because of ARIA risk for the latter, whereas the number of clinics visits would be similar.

### Caregiver burden

#### Estimated caregiver time use

**Figure 1:**
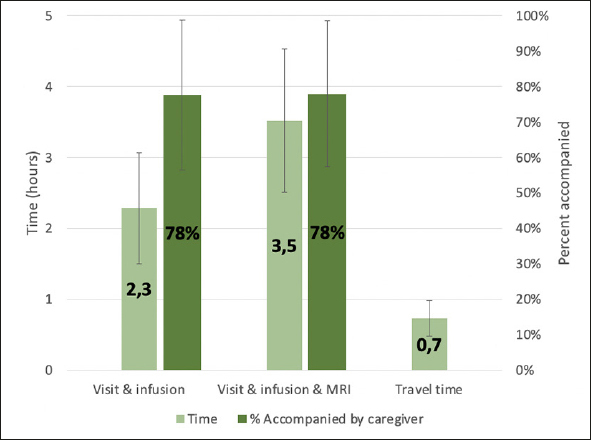
Survey results on caregiver time use

The average reported time estimate for an office visit and infusion was 2.3 hours, with an average of 78% of patients reportedly being accompanied by a caregiver (See Figure 1). The average reported time estimate for an office visit, infusion, and MRI was 3.5 hours, with an average caregiver accompaniment rate of 78%. Round trip travel time was 44 minutes (0.7 hours) on average.

#### Implied cost of lost caregiver time

The valuation of the lost caregiver time is displayed in Table 2. As with direct medical cost, going from intravenous infusions every two weeks to every four weeks approximately halves caregiver burden (52%). The time-limited treatment implies 62% fewer office visits than the chronic treatment that is administered every for weeks, but reduces estimated caregiver burden by 71% because the follow-up visits after treatment end are considerably shorter than the dosing visits. Patterns of caregiver burden for the subcutaneous and oral treatments mirror those observed for direct medical cost: subcutaneous injection and oral application would have 10% and 8%, respectively, of the caregiver burden of a treatment for chronic use and bi-weekly application and 67% and 50% of that for the fixed duration treatment.

### Effect on preservation of treatment value

**Figure 2:**
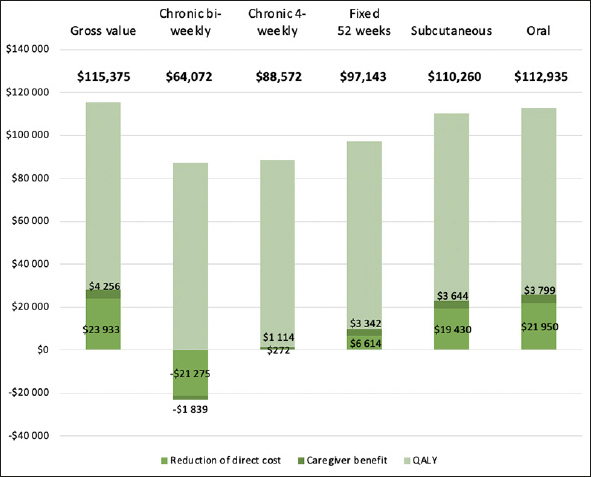
The preservation of treatment value (in bold) in terms of cost offset and caregiver benefit

The effect of differences in medical cost and caregiver burden on the preservation of treatment value for each treatment is presented in Figure 2. As both components exceed the corresponding gains in the case of the chronic bi-weekly infusion treatment, the preserved value would consist of 73% of the valuation of QALY gains or 56% of the overall value, whereas administering the infusion every four weeks would retain 77% of the overall value because of small net reductions of medical cost and caregiver burden. Limiting treatment to 52 weeks would increase the share of preserved value to 84%. Both the subcutaneous and the oral treatment are projected to preserve almost the entire generated value (96% and 98%, respectively).

### Sensitivity analysis

**Figure 3:**
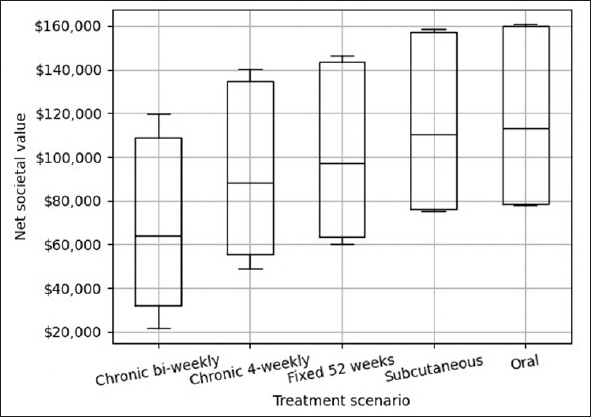
Results from sensitivity analysis

Figure 3 shows the results of the sensitivity analysis that reflects the uncertainty in estimated net value of each of the five treatment scenarios in box plots. The boxes cover the range between the 25th and 75th Confidence Intervals and the lines in the middle the median estimates, which correspond to the point estimates in Figure 2. The results follow the same patterns of differences in net value between the five treatment scenarios, as shown above, but illustrate that the net value will depend on assumptions for treatment effect size, medical cost and caregiver burden.

## Discussion

Our analysis of five hypothetical disease modifying treatment for Alzheimer’s disease showed that the preservation of treatment value is sensitive to the number of clinic visits for infusion delivery, evaluation, and monitoring. The chronic bi-weekly treatment, with the greatest number of clinic visits, would reduce gross treatment value by almost half (44%), while treatment via oral application, with the least number of clinic visits, would preserve 98% of the gross treatment value. The sensitivity analysis showed that the pattern of value retention is robust against changes assumptions for treatment effect size, medical cost and caregiver burden.

Our findings are consistent with a study by Boustani et al. that used a Markov state-transition model to compare the cost-effectiveness of three treatment strategies for a hypothetical DMT started at MCI: continuous treatment until progression to severe AD, fixed-duration treatment for 18 months, and treatment discontinuation for 40% of patients at 6 months (8). The incremental cost compared to standard care from the health care sector perspective was $612,354 per QALY and $157,288 per QALY for the continuous and fixed-duration treatment respectively, a 74% relative reduction in cost for the fixed-duration treatment. A similar study by Ross et al. comparing the cost-effectiveness of aducanumab (continuous) and donanemab (fixed-duration), while using different inputs than Boustani et al., found that the incremental cost relative to standard care from the health care perspective was $981,000 per QALY for aducanumab, versus $193,000 per QALY for donanemab, or a 80% reduction in incremental cost for donanemab (19). Not accounting for potential differences in prices, we found a 68% lower total treatment burden for the fixed duration treatment compared to the chronic treatment administered at the same frequency.

Our study also suggests substantial caregiver burden involved in the administration of DMTs, as it has been observed, for example, in oncology (20). The survey of AD treatment sites found that clinic visits for infusion delivery and examination take about 3 hours on average including the driving time, or 4 hours when the visit also includes an MRI. In addition, treatment sites reported that around four out of five patients (78%) were accompanied by a caregiver for clinic visits. Our finding that patients even at early stages of dementia have substantial caregiving needs is supported by previous research. Other studies have estimated the time caregivers spend caring for patients with MCI. For example, Robinson et al. analyzed a US-based prospective cohort study of patients with early AD (N=1,327) that asked caregivers to estimate the time they spent caring for the patient or the time lost from work due to caregiving (taking the higher of the two), and reported that caregivers spent approximately 84 hours per month caring for patients with MCI (12). Another study by Ryan et al. surveyed caregivers of people with mild dementia, MCI, and normal cognition (N=124) about their service needs, neuropsychiatric symptoms, and functional abilities. They found no significant differences in services needs between the mild dementia and MCI groups, whereas both had higher service needs than individuals with normal cognition, suggesting caregiving needs are already considerable at the MCI stage (21). The aforementioned study by Prados et al. (15) also estimated a limited reduction of caregiver burden from a DMT because of the high baseline burden and slow increase with disease progression.

Our study adds to this literature by cautioning that the time, which caregivers spend to take their charges to clinics for treatment, might exceed the time that they gain from reduced disease progression. Our results suggest that DMTs that require fewer clinic visits have the potential to significantly lower caregiver burden, which could reduce caregiver stress, depression, and chronic health conditions (11).

### Limitations

The findings of this study should be considered in the context of its limitations. Firstly, this analysis is a stylized comparison of hypothetical treatments to isolate the effect of treatment burden on treatment value rather than a comparative analysis of actual treatments, which would have to account for differences in effect size, adverse events, and inclusion and exclusion criteria. In addition, chronic treatments might not be used for the entire period until progression to moderate dementia or at least not at the same interval, and time-limited treatments may need more retreatment doses than assumed. Secondly, we disregard potential differences in rates of treatment discontinuation, which could affect the estimates of gross treatment value and treatment burden. Thirdly, our estimates for caregiver burden are based on a survey of clinic staff rather than actual time use data. Finally, our analysis may underestimate caregiver burden of subcutaneous and oral treatments, as we do not consider the time caregivers might spend on injecting treatment and refilling prescriptions.

## Conclusion

This study is the first to estimate the potential impact of treatment burden on the value of hypothetical DMTs with varying modes of administration and point to the need for less complex treatments that require fewer clinic visits. Future research should develop and evaluate models that support caregivers in their new role of managing treatment delivery.

## Notes

*Ethical standards:* As the study did not constitute human subjects research per U.S. federal regulations (45 CFR 46, 102(f))20, it was exempt from IRB review, consent requirements and registration.
